# Outcomes of an Advanced Epic Personalization Course on Clinician Efficiency through Use of Electronic Medical Records: Retrospective Study

**DOI:** 10.2196/68491

**Published:** 2025-06-20

**Authors:** Junye George Chen, Hao Xing Lai, Shi Min Wong, Terry Ling Te Pan, Er Luen Lim, Zi Qiang Glen Liau

**Affiliations:** 1 Department of Orthopaedic Surgery, Yong Loo Lin School of Medicine, National University of Singapore, Singapore, Singapore; 2 National University Polyclinics Headquarters, Singapore, Singapore; 3 Department of Anaesthesia, Ng Teng Fong General Hospital, Singapore, Singapore; 4 Department of Emergency Medicine, National University of Singapore, Singapore, Singapore; 5 Department of Orthopaedic Surgery, National University Hospital and Alexandra Hospital, NUHS Tower Block, Level 11, 1E Kent Ridge Road, Singapore, 119228, Singapore, 65 67722100

**Keywords:** medical informatics, electronic medical records, EMR, electronic medical records personalization, Epic, electronic health records, EHR, clinician efficiency

## Abstract

**Background:**

Since Singapore’s first migration to *Epic* in 2022, we have been conducting an advanced *Epic* personalization course twice a year for health care professionals with at least 3 months of experience using the system. Electronic medical records education is an under-recognized pillar in reducing health information technology-related stress and clinician burnout.

**Objective:**

The intent of the course is to improve clinician efficiency through customization and personalization of *Epic* interfaces. We hypothesized that compared to their colleagues, trained clinicians would demonstrate significant quantitative improvements in use of the *Epic* system after our course.

**Methods:**

We performed a retrospective analysis from July 2022 to January 2024, including 17 clinicians among 77 individuals who attended our course. Recruitment was done through digital mailers sent out via the local hospital announcement channels. Interested clinicians were able to register for our course via the National University Of Singapore website. Our one-day course involves physical lessons and interprofessional, case-based discussions emphasizing a wide range of high-yield *Epic* functionalities with practice exercises, such as drafting referral letter templates. Three months of pre- and postcourse *Epic* usage statistics of the trained clinicians were retrieved based on aggregate data provided by *Epic* Singapore. Performance metrics included documentation length, time spent in *Epic* functionalities and use of SmartPhrases, order sets and preference lists.

**Results:**

At three months post-course, documentation length decreased by 45.8% (711.8 characters) compared to a 22.2% (126.4 characters) increase among controls. Trained clinicians demonstrated a 2.47-fold increase in use of order sets from 16.2% to 40%, and a 49.9% increase in orders from preference lists after the course, from 35.1% to 52.6%. In total, trained clinicians demonstrated a 1.8-fold increase in combined use of orders from preference lists or order sets after the course, from 51.4% to 92.6%. The number of SmartPhrases created by trained clinicians was 5.64 times higher than among controls, in addition to a 5.57-fold higher use of Quick Filters than controls. Moreover, time in the Chart Review section per day decreased by 29.3% (4.6 min) among trained clinicians versus an increase of 14.6% (2.8 min) among controls. Compared to controls, trained clinicians spent 36.7% (219.6 min) less time in the *Epic* system per day, 56.6% (29 min) less time on Notes per day, and 57.5% (10.7 min) less time on orders per day.

**Conclusions:**

Overall, trained clinicians demonstrated more efficiency in their use of *Epic*, with reduced time spent across various functionalities. Increased use of Smart Tools, including SmartPhrases and Quick Filters was also observed among trained clinicians, which indicated efficiency in reducing total usage time. These findings demonstrated broad improvements across multiple physician efficiency metrics among participating clinicians of our advanced *Epic* personalization course. These gains may contribute to improved mental health among health care professionals and enhanced productivity within health care systems.

## Introduction

### Background and Significance

Singapore’s public healthcare system is organized into three clusters, namely SingHealth, National Healthcare Group (NHG), and the National University Health System. Collectively, these three clusters manage 26 polyclinics, 12 acute general hospitals, and 12 community hospitals, with further expansion in the pipeline to meet the increasing demands of an aging population and chronic diseases [1]. As of 2023, the health workforce includes 17,466 practicing physicians and 38,834 registered nurses [2].

Digitalization of Singapore’s health care system is a strategic initiative, with the Ministry of Health collaborating closely with all three regional clusters to establish an integrated digital health ecosystem. Since 2022, the Ministry of Health has gradually implemented the Next Generation Medical Record system, powered by *Epic* Systems, in NHG and National University Health System institutions, with plans for broader adoption. *Epic* Systems is the most widely used electronic medical record (EMR) system globally [3] and was ranked as the number one EMR system in the United States for the 13th consecutive year in 2023 [4]. The adoption of EMRs, including *Epic*, is mandatory in Singapore’s public health care institutions to establish standardization, data interoperability, and enhanced patient safety [5].

*Epic*’*s* EMR platform offers a diverse array of integrated functions that have been shown to enhance clinical workflows, improve patient safety, and support quality improvement efforts across health care systems [6]. For example, the 2023 KLAS Arch Collaborative survey of more than 35,000 clinicians found that *Epic*’*s* automation, real-time reporting, and interoperability features were strongly associated with greater clinician satisfaction and reduced documentation errors [7]. Embedded safety features such as centralized patient data and barcode medication administration help to accelerate patient recoveries and avoid medical errors, thus routinizing safe evidence-based practices and cost-effective preventive medicine [8]. As *Epic* becomes more commonplace [9], emerging technologies including artificial intelligence–powered scribes may further reduce documentation burden and enhance clinician-patient interaction [10,11].

In response to the recent transition to *Epic*, there is a recognized need for enhanced skills-upgrading and peer-based learning initiatives to optimize system integration among health care professionals [12]. A 2025 scoping review of 90 academic studies highlighted that successful EMR implementation requires collaboration, communication, and the development of user competence through structured training and ongoing support [13]. Health care provider buy-in to *Epic* introduces a culture shift, as new EMRs change the way people communicate, prioritize, delegate tasks, and work together [14,15]. Continuous professional development in EMR usage is strongly encouraged and, in many cases, required to ensure clinicians remain proficient and adaptable as the system evolves in Singapore and more hospitals adopt the NGEMR [16].

Reaffirming this commitment, the Yong Loo Lin School of Medicine at the National University of Singapore has introduced a dedicated Continuing Education course titled “How to make the most out of Epic.” Being the first course of its kind in Singapore, it is tailored for doctors, nurses, and allied health professionals who have at least three months of experience using the *Epic* system. This advanced personalization course, conducted biannually as a one-day on-campus session, is designed to deepen users’ understanding of *Epic*’s functionalities and promote optimal use in clinical practice. The curriculum emphasizes interprofessional collaboration through case-based discussions, allowing participating clinicians to explore real-world scenarios and share evidence-based practices that can drive improvements in patient care.

### Objective

The aim of our retrospective study is to evaluate the longitudinal effect of an *Epic* learning course on improving the informatics competency of clinicians in Singapore over a six-month period, with the hypothesis that *Epic* training would translate into greater capacity for efficiency and productivity.

## Methods

We followed the STROBE guidelines in conducting this review.

### Study Design and Sample Selection

Our course was advertised on the National University Of Singapore website [17] and through electronic mailers via hospital announcement channels. Interested clinicians were able to register for the course via a link on the website.

The “How to make the most out of Epic” official course date was defined as the index date, and data for each clinician was retrospectively collated for the 3 months preceding the index date (ie, preindex period) and for 3 months after index (ie, postindex period). Clinicians were included in the final analysis if they fulfilled the following criteria: (1) ≥21 years of age on the index date; (2) employed as health care clinicians at a health care institution in Singapore (3) ≥3 months of experience using *Epic* as an EMR system at work; and (4) achieved 100% attendance in the “How to make the most out of Epic” course.

A total of 77 individuals including clinicians, nurses, allied health professionals, and administrative executives participated in the course. However, data were available for only 17 clinicians, comprising 15 medical doctors and 2 nurse clinicians. These clinicians were case-controlled against their colleagues from the same clinical departments who did not attend “How to make the most out of Epic” course The analyzed clinicians included attendees from three iterations of the same course, offered biannually, with index dates of October 4, 2022, March 7, 2023, and October 10, 2023, respectively.

### Training Contents

The one-day course involving physical lessons and interprofessional case-based discussion sought to “personalize and make *Epic* work for the participants.” A wide variety of high-yielding *Epic* functionalities were emphasized, with accompanying practice exercises, including but not limited to performing QuickWin features such as Chart Searches, using keyboard shortcuts, creating and editing SmartPhrases, customizing personal preference lists, setting up order sets, and creating referral letter templates. The course is stackable; completion of three other Informatics courses within a 24-month period contributes to an Executive Certificate in Healthcare Improvement via Informatics, accredited by the National University of Singapore, and recognized across Singapore’s health care sector for relevant informatics and quality improvement skills.

### Data Source

This retrospective, descriptive study used administrative signals data from *Epic*, collected between July 2022 and January 2024. The Epic database consists of aggregate, system-captured metrics pertaining to *Epic* use.

### Ethical Considerations

This study was approved with exemption by our Institutional Review Board (NHG DSRB Ref: 2024‐3739), without the need for informed consent. Privacy and confidentiality protection were maintained by deidentifying the study data. No compensation was provided to the participants during the course of our study. No identification of individual participants was included in any manuscript images or supplementary material.

### Study Outcomes and Variables

The analyzed data included the length of typed phrases, clinicians’ habits in placing clinical orders via *Epic*, time spent using various *Epic* functions, creation of Quick Filters, and use of Chart Search across both pre- and postindex periods. Quantitative data trends were visualized using line graphs generated with Microsoft Excel 2024.

## Results

To put our personalized *Epic* training program into context, it is critical to first understand the architecture and features of the *Epic* EMR system. Within *Epic*, the clinicians used a set of customizable instruments to improve their capacity to document, order, and review patient information. For example, the Chart Search function allows immediate retrieval of desired terms or data points within a patient’s record. Quick Filters allow clinicians to efficiently narrow down encounters, notes, or test results, permitting targeted review. Keyboard shortcuts and navigation aids such as the “pick and stick” technique expedite movement through the system and reduce repetitive tasks.

*Epic* also structures clinical workflows across several modules. Chart Review is a workspace where clinicians can browse a patient’s complete medical record, including previous encounters, laboratory results, imaging, and documents in one place. The Clinical Review module is a summary dashboard that shows current clinical information such as problem lists, medications, allergies, and pending tasks, to support timely clinical decision making. The Basket is an integrated communication and task management hub within *Epic,* which hosts notifications, test results, cosignature requests, and other messages requiring clinician attention.

In addition to these core functionalities, *Epic* offers several features specifically designed to improve documentation efficiency and workflow. SmartPhrases are user-defined templates or phrases that can be quickly inserted into clinical notes to standardize and accelerate documentation. Personal preference lists further allow clinicians to tailor frequently used orders or documentation elements for rapid access, while order sets enable the bundling of related orders and documentation templates for common clinical scenarios. Collectively, these features affect the length and structure of typed phrases, clinicians’ ordering habits, and time allocation across *Epic* functions. Thus, they are valuable parameters which allow us to evaluate the effectiveness of targeted *Epic* training interventions.

Based on the collective experience of the authors and course instructors, many clinicians initially find the advanced personalized functions such as SmartPhrases, preference lists, and order sets, challenging to master. Consequently, these components were a primary focus of our advanced *Epic* training curriculum. Following the completion of this specialized course, three major themes emerged in the EMR use patterns: improvements in documentation workflow, enhanced time management, and increased efficiency in order management.

**Figure 1.**
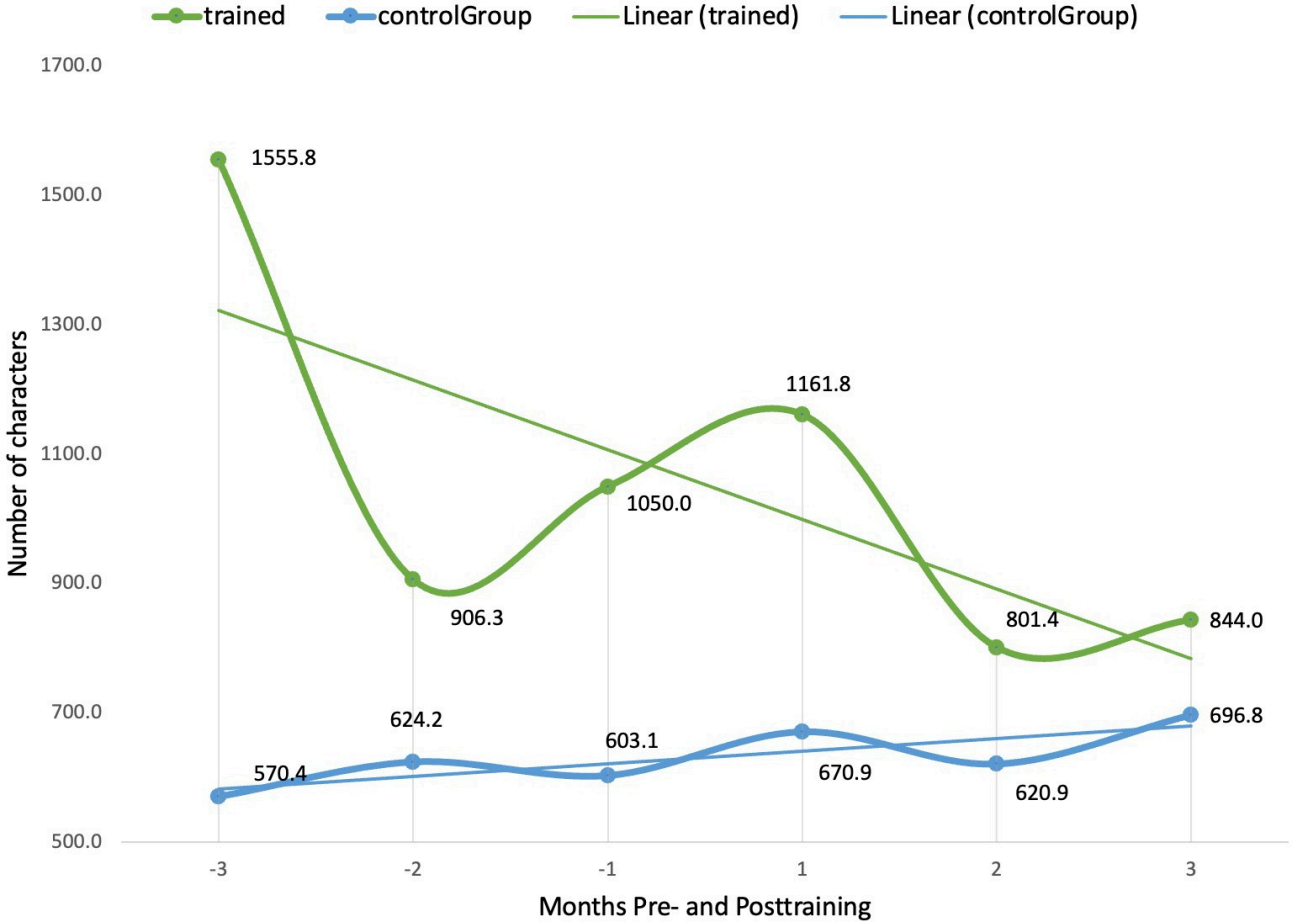
Comparison of documentation length in *Epic* (number of characters) between trained clinicians and control participants, 3 months pre- and posttraining.

First, the trained cases recorded a decreased burden of documentation compared to the control participants, likely due to an enhanced documentation workflow. When comparing the 3 months post- and precourse, respectively, the documentation length, measured by the number of characters, decreased by 45.8% (711.8 characters) among trained clinicians, versus an increase of 22.2% (126.4 characters) among control participants (Figure 1). Similarly, progress note length was also found to decrease by 46.9% (665.7 characters) among trained clinicians, as opposed to an increase of 34.9% (326.1 characters) among control participants. Concomitantly, we observed that the number of SmartPhrases created by trained clinicians was significantly higher at 5.64 times more than that of the controls at 3 months postcourse. SmartPhrases, being precurated abbreviations or sentence templates, reduce manual typing and contribute directly to improved documentation efficiency, contributing to the reduction in documentation length and typing burden.

**Figure 2.**
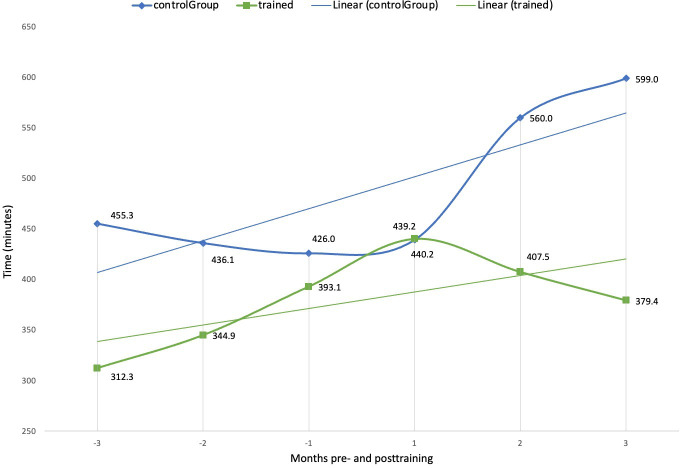
Comparison of time in epic system per day (min) between trained clinicians and controls 3 months pre and posttraining.

**Figure 3.**
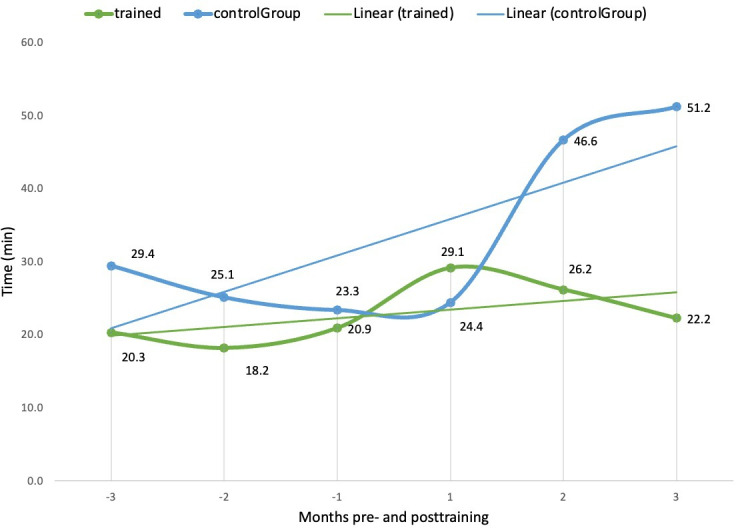
Comparison of time in notes per day (min) between trained clinicians and controls 3 months pre and posttraining.

**Figure 4.**
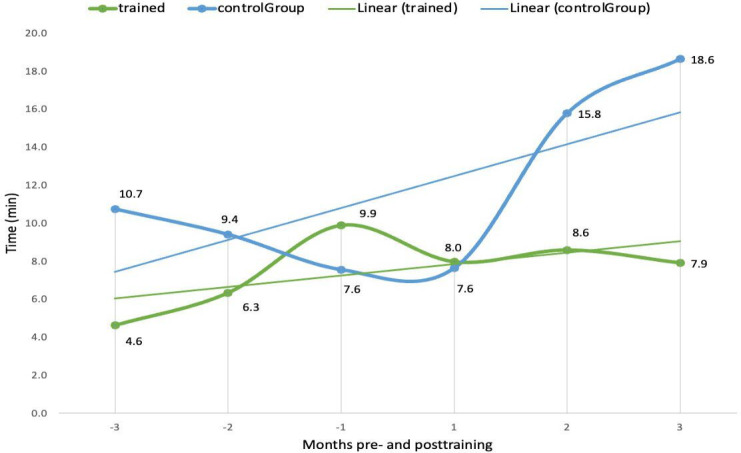
Comparison of time in orders per day (min) between trained clinicians and controls 3 months pre and posttraining.

The second theme revolves around reduced active system use time, possibly as a result of better use of various functionalities of Epic. On average, trained clinicians spent 36.7% (219.6 min) less time using the Epic system per day than controls at 3 months postcourse (Figure 2). Time spent in the Notes module per day was 56.6% (29 min) lower for trained clinicians than controls at 3 months post-course (Figure 3). Additionally, trained clinicians spent 53.4% (19.4 min) less time in Clinical Review and 57.5% (10.7 min) less time on orders per day compared to the controls (Figure 4), while the use of Basket module, time per day remained similar between both groups. Moreover, time in the Chart Review module per day decreased by 29.3% (4.6 min) for trained clinicians, while control participants showed an increase by 14.6% (2.8 min). These data suggest that advanced training facilitated more efficient navigation and use of *Epics*’ module, allowing clinicians to devote less time to routine EMR tasks.

**Figure 5.**
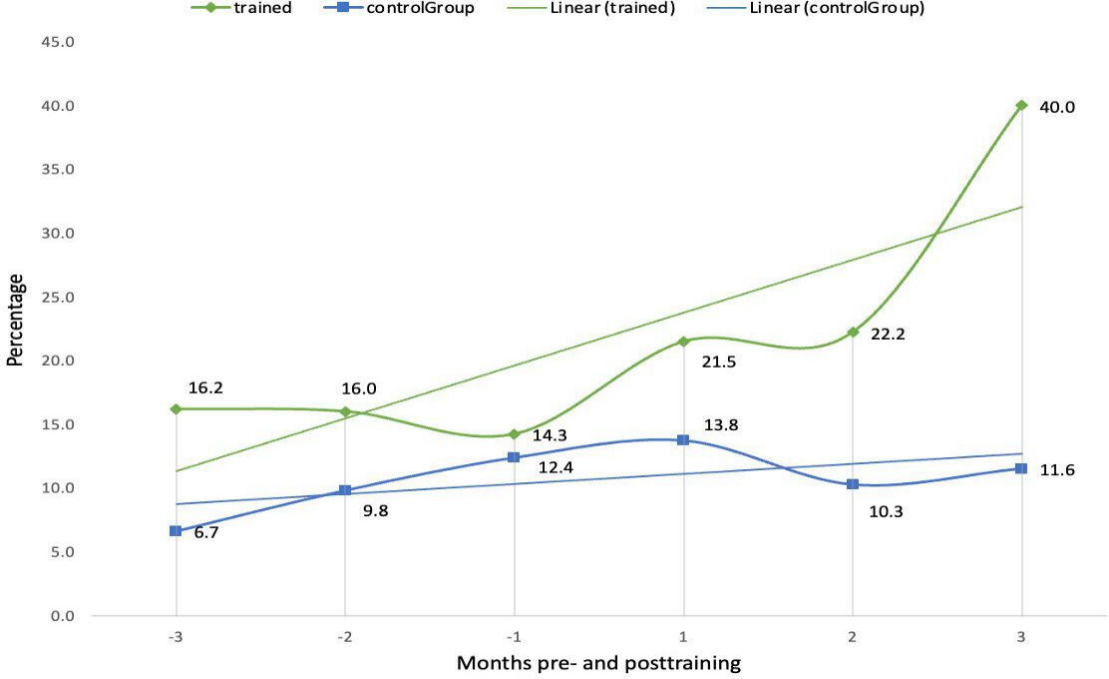
Comparison of percentage of orders from order set (%) between trained clinicians and controls 3 months pre and posttraining.

**Figure 6.**
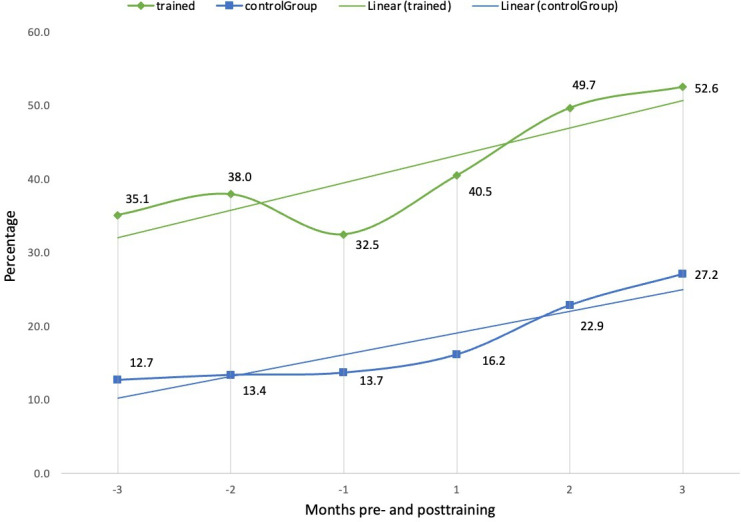
Comparison of orders from preference lists (%) between trained clinicians and controls, 3 months pre- and posttraining. -

The third key finding centred on increased efficiency in order management, particularly through the customization and use of *Epic’s* advanced ordering tools. Trained clinicians demonstrated greater resourcefulness in tailoring their use of preference lists and order sets. Trained clinicians had a 2.47-fold increased use of order sets from 16.2% to 40% (Figure 5) and a 49.9% increase in orders from preference lists after the course from 35.1% to 52.6% (Figure 6). In total, trained clinicians had a 1.8-fold increased use in orders from preference list or order sets after the course from 51.4% to 92.6%. Additionally, trained clinicians used Quick Filters 5.57 times more compared to controls. Preference lists allow clinicians to save frequently used orders for medications or investigations, while order sets extend this functionality by bundling multiple preference lists. The increased adoption of these customized interfaces significantly streamlined ordering processes and highlights the role of targeted training in reducing both cognitive and time burdens associated with electronic order management.

## Discussion

### Principal Findings

This study aimed to evaluate the impact of an advanced *Epic* EMR training course, “How to make the most out of Epic,” on clinician efficiency and workflow in Singapore, within the broader context of competency-based medical education and the growing emphasis on medical informatics [18]. Our findings demonstrate that trained clinicians who completed the course exhibited significant improvements in time management and efficiency across key ambulatory metrics, including documentation, order entry, and clinical review. These outcomes align with the course’s objective of bridging interprofessional collaboration and enhancing the practical use of EMR smart features, supporting the integration of informatics competencies into continuing medical education at the Yong Loo Lin School of Medicine, National University of Singapore.

Our results revealed that structured EMR training empowers clinicians to optimize the use of advanced features within *Epic* and harness its potential to improve the quality of health care, consistent with *Epic* being the most studied EMR system for its robust features [19]. Similar training programs conducted in the United States, where *Epic* originated, have shown remarkable gains relating to clinician proficiency [20]. For example, Hollister-Meadows et al [21] found that 133 *Epic*-trained clinicians sustained an average increase of 16 patient consultations per 30-day reporting period, whereas 33 untrained clinicians experienced a decrease of 8 patient consultations [21]. Novel approaches include peer-based training at the Vanderbilt University Medical Center [22], foster a synergistic community to help clinicians overcome the EMR learning curves. Likewise, our course brought together doctors, nurses, and allied health specialists to strengthen interprofessional collaboration workflows using real-world patient examples and scenarios.

Greater mastery of *Epic* provides multifarious benefits for patient care, including standardized aggregation of electronic health information, effective use of decision support tools, and enhanced integration of software buttons with clinical workflows [23]. The findings from our retrospective descriptive study are consistent with that of department-focused “EHR thrive” training conducted by Livingston and Bovi [24], in which trained clinicians demonstrated improved efficiency in time management across documentation, basket management, and clinical orders. Cumulatively, increased utilization of Smart Features within various ambulatory metrics (ie, Notes, Orders, Chart Review, Clinical Review) reduces unnecessary screen time, increases productivity within the health care delivery system, and optimizes integrated care for the patients. As a result, clinicians may devote more time per consultation in direct patient interaction and shared decision-making, rather than “desktop medicine.” Further, EMR training and education is often an under-recognized yet important component in reducing Health information technology–related stress and clinician burnout [25], thereby reducing the risk of medical errors. Expanded *Epic* research capabilities into patient-based and health services databases further contribute to improvement in quality of care [26].

Despite these promising results, several limitations must be acknowledged. Our findings are limited in generalizability due to the retrospective study design and small sample size (n=17), which was affected by recruitment barriers such as mandatory full-day attendance and course fees. Further, only aggregate data was analyzed for confidentiality, which precluded statistical significance testing; demographic information and EMR use duration were unavailable, as these data were not captured by *Epic*. Furthermore, we were unable to assess the provider efficiency profile and *Epic* proficiency scores, which are valuable surrogate measures of EMR training effectiveness as demonstrated by Livingston and Bovi [24]. Inclusion of these metrics would have enabled adjustment for intermonth variability related to clinical workload. Future studies should consider prospective, randomized recruitment, and participant stratification to adjust for confounders such as clinician age, seniority, and previous duration of *Epic* usage.

### Conclusion

This preliminary study demonstrates an example of university-led, interprofessional *Epic* training aimed at improving clinician competencies and productivity with EMR interaction. The findings showed significant improvements in efficiency across metrics such as Notes, Chart Review, Clinical Review, and Orders. This evaluation also highlights the benefits of using Smart Tools to improve time management during patient consultation, consistent with literature. Becoming “Epic Smart” and “Computer Smart” extends beyond individual clinician performance. As health care transitions toward digital transformation, training clinicians and students in advanced EMR skills is key to adapting to changing care models and using data-driven decision support. Health care clinicians are encouraged to participate in advanced EMR training programs. Continued investment in informatics education will be crucial to developing a strong workforce capable of meeting the challenges and opportunities of today’s technology-enabled health care.

## Supplementary material

10.2196/68491 Checklist 1 STROBE (Strengthening the Reporting of Observational studies in Epidemiology) Checklist.

## Abbreviations

EMR: electronic medical record
NHG: National Healthcare Group
